# Point-of-care diagnosis of cervical cancer: potential protein biomarkers in cervicovaginal fluid

**DOI:** 10.55730/1300-0152.2608

**Published:** 2022-04-01

**Authors:** Büşra KÖSE, Özgüç TAKMAZ, Mete GÜNGÖR, Ahmet Tarık BAYKAL

**Affiliations:** 1 Department of Biochemistry and Molecular Biology, Institute of Health Sciences, University of Acıbadem Mehmet Ali Aydınlar, İstanbul, Turkey; 2 School of Medicine, Department of Obstetrics and Gynecology, University of Acıbadem Mehmet Ali Aydınlar, İstanbul, Turkey; 3 Acıbadem Maslak Hospital, Department of Obstetrics and Gynecology, İstanbul, Turkey; 4 School of Medicine, Department of Medical Biochemistry, University of Acıbadem Mehmet Ali Aydınlar, İstanbul, Turkey

**Keywords:** Cervical cancer, biosensor, cervicovaginal fluid, proteomics, protein biomarker

## Abstract

Cervical cancer (CxCa) is preventable and treatable via vaccination and screening. Cervicovaginal fluid (CVF) represents the physiological components of the female genital tract. These components are suitable to be utilized for clinical purposes, therefore, making CVF a suitable material for disease screening approaches.

Due to high false-negative result rates and low attendance of current expensive routine CxCa screening methods, it has become more important to develop a point-of-care (POC) screening method that every single woman could reach worldwide. For this purpose, various self-usage apparatus have been developed for screening of the human papilloma virus (HPV) infection.

Furthermore, due to the low specificity of HPV tests and the high clearance rate of HPV infections, many patients undergo overtreatment. Since proteins play an important role in cellular process and carcinogenesis, it is appropriate to use proteins in a simple screening test for the detection of carcinogenesis.

In this article, POC screening tests and the studies of discovery of CVF protein biomarkers will be overviewed to consider the development of a method that can be used for the rapid and conceivable screening method of CxCa.

[Table t2-turkjbiol-46-3-195]

**Table t2-turkjbiol-46-3-195:** 

Contents	
Point-of-care diagnosis of cervical cancer: potential protein biomarkers in cervicovaginal fluid	1
1. Introduction	6
2. Current CxCa screening methods used worldwide	7
3. Overview of self-sampling and POC device technology for HPV infection and CxCa screening	10
4. A body fluid in clinical diagnosis: cervicovaginal fluid	15
5. Proteomics studies and potential protein biomarkers from CVF for CxCa	18
6. Conclusion	23
References	**Hata! Yer işareti tanımlanmamış**.

## 1. Introduction

The cervix, consisting of the ectocervix and endocervix, is the muscle where the uterus opens into the vagina. The endocervix is composed of columnar mucus-secreting cells, while the ectocervix consists of squamous epithelial cells. Under unfavorable conditions, the squamous epithelial cells in the ectocervix undergo various morphological and metabolic changes. The area where such transformation occurs is referred to as the “transformation zone” and is where carcinogenesis begins (Arbyn et al., 2020). Typically, invasive tumors develop after the premalign lesions turn into neoplastic lesions (de Martel et al., 2017). Regarding cervical transformation, this process usually takes between 2 and 15 years and results more commonly in (approximately 90%) squamous cell carcinoma than in (approximately 10%) adenocarcinoma (Arbyn et al., 2020). Cervical cancer (CxCa) is the fourth most common female cancer worldwide, with approximately half a million new CxCa cases diagnosed every year (Arbyn et al., 2020). In 2018, 311 thousand women died of CxCa worldwide according to the World Health Organization (WHO) reports (Arbyn et al., 2020). The most common (99.99%) etiology of CxCa is HPV infection (Arbyn et al., 2020), which is transmitted via the sexual route.

Management of cervical cancer screening follows the current guidelines. Since these cervical pathologies usually do not cause any symptoms, the cervix is routinely screened through Pap-smear test, and HPV genotyping for sexually-active female adults, regardless of any complaint. However, due to the frequent false-negative results of Pap-smear tests for screening CxCa, and the reduced availability of medical follow-up in low- and middle-income countries (LMICs), the development of self-screening tests is critical for improving the health of women worldwide. In addition, HPV infection has a high self-clearance rate, and in most cases, HPV positivity does not predict cancer development leading to overtreatment in HPV-positive patients. Therefore, a novel screening method with novel biomarkers for the detecting and prognosis of CxCa is critical to improve the wellness of women worldwide (Castle et al., 2016; de Martel et al., 2017; Abbas et al., 2019; Silver et al., 2020). Concerning this, studies for the investigation of new alternative screening approaches have been conducted for the last 20 years (Zegels et al., 2018).

## 2. Current CxCa screening methods used worldwide

HPV is the most common viral infection of the female reproductive tract, and HPV is mainly transmitted through sexual intercourse. An average adult has an 80% risk of contracting the HPV infection by the age of fifty. Every year, 570,000 cases among females and 60,000 cases among males can be attributed to HPV infection among cancer cases. Despite advances in HPV vaccination, vaccines cannot provide full protection due to the diversity of the virus in nature. To date, more than 170 HPV types have been identified; 40 of which cause various infectious diseases and 20 of which settle in genital tissues in humans. Vaccines are developed only against the most common and risky types of HPV.

In particular, vaccines have been developed against HPV-6, 11, 16, and 18 types, which have most commonly been detected in CxCx cases (Silver et al., 2020). Although the HPV vaccine has 100% protection against the antigen types contained in children and adults who have never been exposed to the virus, it may also be effective in infected individuals (Silver et al., 2020). The vaccine protection is known for a maximum of 10 years. Therefore, an effective competent screening method for cervical cancer is required, which is a disease caused by this common infection and is highly treatable (Castle et al., 2016; de Martel et al., 2017; Abbas et al., 2019; Silver et al., 2020).

**Figure 1:**
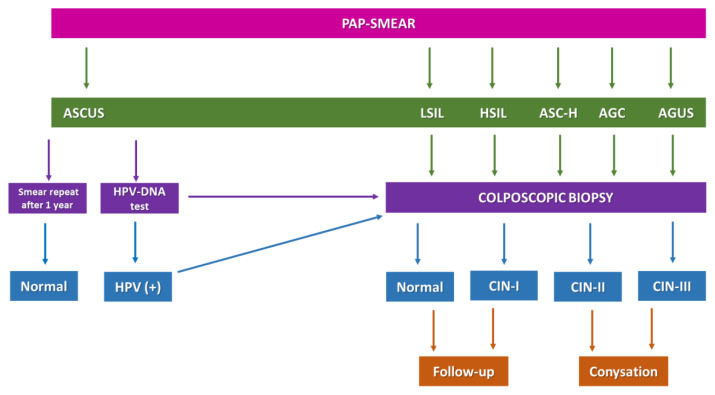
Cervical screening. Pap-smear test results can be normal, unclear, or abnormal. Abnormal results were given in the figure. ASCUS is the most common abnormal result. Mostly, Pap-smear test is demanded again after 1 year for ASCUS result. But, if HPV infection is present with the ASCUS, colposcopy is performed. When the cervical screening test results are abnormal, usually colposcopy is demanded. Colposcopic biopsy results reveal the stage of neoplasia at the histological level. If there is a neoplasia development in the tissue, curettage is performed to purify the cervix from them.

**Figure 2:**
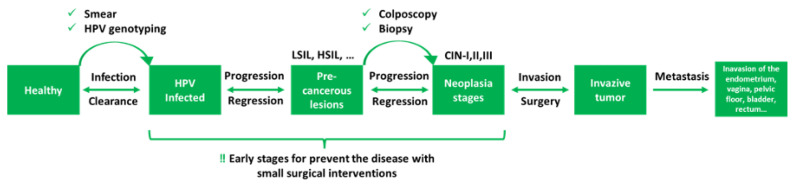
Transformation of the cervix from HPV infection to cancer of the cervix. Developmental phases of cervical carcinogenesis: Most of the HPV infections spontaneously show regression within two years. However, regarding precancerous lesions, persistent high-risk HPV infection, if stays untreated, may progress to high-grade lesions and metastasis.

Current diagnostic methods include Pap-smear test (cytological examination of the cervix external layer), HPV genotyping (based on (PCR) polymerase chain reaction), colposcopic imaging, cervicographic imaging, speculoscopic imaging, needle biopsy, and cone biopsy. Pap-smear test detects abnormal cells, produced by the infected transformation zone, in cervical smear samples (Schiffman et al., 2007). Pap-smear screening detects and identifies the HPV-associated lesions (Figure 1) as “atypical squamous cells of undetermined significance” (ASCUS), “low-squamous intraepithelial lesions (LSIL), high-squamous intraepithelial lesions” (HSIL), “atypical squamous cells, although it cannot exclude HSIL” (ASC-H), atypical glandular cells (AGC), and “atypical glandular cells of unknown significance” (AGUS) as the most common results (Schiffman et al., 2007). The results of the Pap-smear and HPV genotyping test (which those two tests are frequently referred to as “cotest” in many countries) indicate if the colposcopic biopsy is required to devise a therapeutic strategy (Figure 2). Usually, immunostaining methods are applied during the colposcopic biopsy examination to isolated basal cells or superficial layers of the tissue. Neoplasia stages are diagnosed according to the staining as negative, focal, or diffused based on cytoplasmic and/or nuclear staining. Histopathological analysis of colposcopic biopsy samples reveals the presence of cervical intraepithelial neoplasia, which is graded as 1, 2, or 3 (low/intermediate/high-level neoplasia: (Figure 1) CIN-I, -II, and -III) stages (Schiffman et al., 2007).

Pap-smear screening has been documented to decrease both the incidence and mortality rates of CxCa (Schiffman et al., 2007; Arbyn et al., 2020). Nevertheless, since it requires specific materials and stains, it is an expensive test that may be beyond the reach of average women in LMICs. Also, it does not ensure the “number needed to treat” (i.e. NNT, a measure is calculated on the inverse of the absolute risk reduction of the cases. It represents the number of real patients who need to be treated, except for those who do not need any medical intervention at that moment, to prevent a bad outcome) and has low reliability (Koonmee et al., 2017). Its sensitivity is 90%, specificity is 70%; confidence interval is 37%–66%; and the false-negative result rate ranges from 15% to 70% (Castle et al., 2002; Vasconcelos et al., 2014; Koonmee et al., 2017). The disadvantages of Pap-smear-only screening lead many countries to HPV-PCR screening which suggests a major economic burden for LMICs.

CxCa screening and vaccination programs are effective in lowering the public risk of developing this disease (Schiffman et al., 2007; Arbyn et al., 2020); however, few of these programs are available to citizens from lower socioeconomic groups, as well as to those with limited education and/or limited resources (Wikström et al., 2011). Major reasons for low attendance rates to screening and vaccination programs include limited access to female health facilities, exorbitant healthcare costs, lack of insurance, time and transportation limitations caused by working conditions, social stigma, moral value judgments, and fear of poor outcomes or pain (Wikström et al., 2011; Sangwa-Lugoma et al., 2011; Bal et al., 2014; Maza et al., 2018; Devarapalli et al., 2018; WHO, 2018).Along with the low attendance to screening and prevention, CxCa incidences are considerably higher in LMICs (Schiffman et al., 2007; WHO, 2018). The underlying reasons for this include high rates of infection and low rates of vaccination, limited hygiene and/or healthcare service access, and frequent polygamy and prostitution (Sangwa-Lugoma et al., 2011). The most possible method for resolving these issues is identifying cervical neoplasia biomarkers and developing a self-screening test, this would be a lifesaver tool especially in those hardship areas. CxCa has a slow progression, therefore a relatively preventable disease, suggesting that it may be possible to eliminate CxCa as a public health problem via widespread vaccination and screening programs (Canfell et al., 2018). Presently, easily accessible methods/devices could be effective in both developed and less developed countries.

## 3. Overview of self-sampling and POC device technology for HPV infection and CxCa screening

Point-of-care (POC) diagnosis is a promising approach for screening certain diseases. The goal of this approach must be to detect disease-related specific biomarkers from easily accessible body fluids that were obtained through self-sampling apparatus. The device must serve as an easy-to-use, inexpensive, and noninvasive method.

Generally, POC devices can be microfluidic electrochemical biosensors; they may contain fluorescent, nanoparticles, or quantum dots technology, or they may consist of simple paper-based microfluidic immunochromatographic tests (Wilson et al., 2006). The type of most common and basic one of the paper-based microfluidic immunochromatographic tests is referred to as the “lateral flow assay” (LFA) which uses urine or blood specimen and works based on the antigen-antibody, or the enzyme-substrate chemiluminescence interactions. LFAs are the easy-to-use technology for low-cost, rapid, portable, and simple devices that are used for detecting anything. LFAs working principle basis on quick immunological reactions in other words, qualitative and quantitative detection of specific antigen-antibodies interaction. LFA-based devices are widely purchased in pharmacies and used anywhere, pregnancy test is an example of that (Wilson et al., 2006). Generally, POC tests can be combined with self-sampling apparatus and can be used either by medical personnel or by patients themselves.

Since many people in LMICs do not have access to full healthcare services, early diagnosis of certain diseases is depend on the POC tests, under those circumstances. Currently, there are numerous POC tests available for the detection of various communicable diseases, especially viral or bacterial infections (Warren et al., 2014; Tay et al., 2016; Vashist & Luong, 2018; Peeling & Mabey, 2010; Chen et al., 2019).

Clinical laboratories offer high-quality diagnostic tests, such as PCR, genome sequencing, blood or urine culture, immunoassays, and mass spectrometry analysis, which are time-consuming, expensive, and rely on sophisticated instruments and well-trained personnel. In contrast, POC tests offer low-cost, portable solutions that are simple to administer and along with providing rapid results, which allows better short-term care, such as glucometers. Furthermore, as the most critical point, routine screening of certain diseases through self-tests emphasizes preventive medicine rather than treatment. Therefore, it has been reported that POC tests provide immediate results, can thus act as a personal indicator for medical intervention (Tay et al., 2016). Consequently, spreading the POC devices worldwide would be a goal of global healthcare.

According to the World Health Organization, POC tests that address disease control needs, especially in developing countries, must meet the “ASSURED” criteria: (1) affordable, (2) sensitive, (3) specific, (4) user-friendly, (5) rapid and robust, (6) equipment-free, and (7) deliverable to end-users (Tay et al., 2016; Chen et al., 2019).

**Figure 3:**
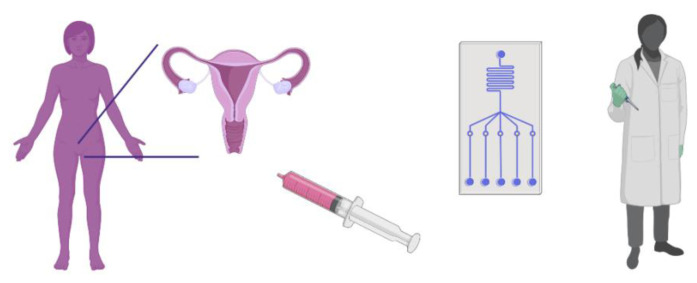
An illustration of the POC screening approach. The intended application of POC device for CxCa screening through CVF: The combination of cervicovaginal lavage aspiration sampling and POC device, rapid screening at physician’s examination room *(created with Biorender.com)*. This method requires neither a high-profile laboratory nor an expert. Since it must result in minutes through the CVF sample easily, the device could be a microfluidic interdigitated capacitor-based biosensor for sensitive detection of multiple molecular biomarkers.

In recent years, several studies have focused on developing a novel sampling method for CxCa screening. Arbyn et al. (2014) showed that self-sampling could provide an increase in attendance at screening programs (Arbyn et al., 2014). Since the mid-2000s, self-sampling apparatus, such as apparatus to collect the urine sample (e.g., Colli-Pee; Vorsters et al., 2014), the brush to obtain a cytological specimen (e.g., Evalyn Brush; Boers et al., 2014), and the apparatus to obtain the vaginal lavage sample (e.g., Delphi Screener; Malone et al., 2020), have been developed for screening the HPV infection. Most of these were reviewed by Othman et al. (Othman et al., 2014). These self-sampling apparatuses have created advances in the development of POC devices for self-screening. The screening test with self-sampling can be performed either by patients in their homes or by doctors in their clinics (Figure 3). Furthermore, there are also certain developments about POC devices for screening different cancer types and/or infectious diseases. Owing to the promising specifications of POC devices, several new approaches for biosensors were reported in the literature. In this review, some of them will have been included briefly.

Raamanathan et al. aimed to build a novel microfluidic system programmed to detect the circular marker CA-125. They constructed a rapid, safe, and precise miniaturized detection system that can be used for ovarian cancer screening (Raamanathan et al., 2012). They compared the results of this system with the enzyme-linked immunosorbentassay(ELISA) methodasthegoldstandard, and they found that the coefficient of determination (R^2^) is 0.97 with a 4% coefficient of variation (CV). However, since the CA-125 marker is also overexpressed in CxCa and endometrial cancer (Calis et al., 2016), as well as many benign conditions the specificity of this system for ovarian cancer screening remains questionable.

Lam et al. developed an electronic, portable, self-usage colposcopy test, called the “POCket colposcope” to monitor CxCa prognosis in 2015 (Lam et al., 2015). This device was evaluated for clinical practice in Tanzania and Peru, reported to be simple to use and capturing a high-quality image of the surface of the cervix. The device was found to have higher sensitivity and specificity rates for discriminating normal/benign cases from CIN stages than those of the eyes of three expert physicians. In LMICs, for the average population, the device may be more accessible and affordable than usual colposcopic examination.

Mohammed et al. reported that valosin-containing protein (VCP) was significantly overexpressed in CIN-II, CIN-III, and CxCa tissues (p < 0.005) than the healthy tissue, and they confirmed its overexpression using both Western blotting and immunohistochemistry (Mohammed et al., 2016). This finding encouraged the group to develop a POC test based on an enhanced lateral flow immunochromatography assay designed to detect VCP in cervical tissues. This POC device was designed to indicate the VCP-positive samples via color change. This POC device was designed to indicate the VCP-positive samples via color change through enhanced magnetic-focus lateral flow immunochromatographic assay (mLFS). Following this design, the same team published a method article describing a three-year longitudinal study evaluating the application of this device in the clinic (Ren et al., 2019). The magnetic-focusing increases the retention time of the target through gold Fe_3_O_4_ core and gold shell-coated nanomagnetic probes labeled with antibodies are specific to the target protein at the reaction zone of the detection. They optimized the prototype for alternative sample types, such as cervicovaginal lavage or liquid cytological specimens, and reported that an ultrasensitive magnetic-focus lateral flow biosensor that detects protein biomarkers in a practical format, capable of detecting as little as 25 fg/mL protein. The sensitivity was found improved by 2 × 10^4^ times compared to the conventional LFA method. Also, they claimed that the target capturing efficiency of this magnetic focusing system is higher than that of conventional LFA systems by up to 10^6^-fold. The device was furtherly tested by analyzing with the clinical samples: The amount of the VCP was detected at 16 pg/mL by the device in the protein mixture, extracted from cervical tissue samples of CxCa patients.

To address the issue of limited financial resources, Inan et al. created a POC device that detects the HPV viral oncoprotein in whole blood using a noninvasive, inexpensive, rapid, and easy-to-perform method (Inan et al., 2017). This test uses an immunoassay and microfilter-based plasma separator for the isolation and quantification of the E7 oncoprotein from HPV-16 to determine the presence of high-risk HPV infections. The area under the curve (AUC) for this system was found to be 0.95 and the test had 94% of sensitivity and 85% of specificity. This customized simple immunoassay test with the microfluidic filter device offers a rapid, noninvasive, low-cost, reliable pretesting tool; and also reduces the burden of current expensive methods for cervical cancer screening in large populations with limited resources.

Another example of HPV self-screening test is nucleic acid amplification-based: Rodriguez et al. developed a POC chip for the risk detection of CxCa, made entirely out of paper and adhesive sheets, making it equipment-free, low-cost, portable, disposable, easy to manufacture, and simple to use. This chip can operate directly with cervical cytological specimens in less than 1 h using isothermal loop-mediated amplification (LAMP) to detect the expression of the E7 oncogene of HPV-16 (Rodriguez et al., 2017).

Zhang et al. compared the immunological paper-based test “OncoE6/E7” with the widely used liquid-based Pap-smear test “ThinPrep”. They found that the OncoE6/E7 test is capable of detecting E6/E7 oncoproteins from HPV-16 and -18 which are the most common types of CxCa risk, in the cytological specimens (Zhang et al., 2018). They showed that its sensitivity was higher (65.5% versus 36.2%), but its specificity was lower (38.2% versus 88.2%) than those of the ThinPrep cytological test for CIN diagnosis.

Appidi et al. offered a simple quantitative screening of CxCa. The method works with “C-ColAur” (Cervical cancer detection using Colorimetric sensing with Au-nanoparticles) which exposes the characteristic color. CVF samples were mixed with HAuCl_4_, followed by ascorbic acid reduction; therefore, this resulted in immediate color changes in the samples. They found that the control gold nanoparticles (formed with healthy samples) were bright blue, while the test gold nanoparticles (formed with cancerous samples) were colorless. They reported that this color changing was due to the formation of Au nanoparticles and the size/shape/interparticle distance-dependent localized surface plasmon resonance (SPR) of those. The detection technique C-ColAur was validated for its sensitivity and specificity, by comparing to Pap-smear test and/or colposcopic biopsy as the gold standards. Out of 42 samples, cancerous samples were identified in 28/27 and healthy samples 14/10 were identified correctly. The technique was reported with a high confirmation rate (approximately 96%) and high sensitivity (of approximately 96%) and specificity (of approximately 87%) for the efficiency of the method for early CxCa screening (Appidi et al., 2020).

## 4. A body fluid in clinical diagnosis: cervicovaginal fluid

Following the development of novel alternative screening methods through POC tests, alternative biological materials for CxCa screening started to be investigated. Concordantly, cervicovaginal fluid (CVF) has a higher quality concerning the context compared to the smear sample. Moreover, CVF collection provides the direct secretions of the cervicovaginal region which comprises the transformation zone. In addition, its collection does not require medical assistance. Accordingly, studies on the CVF content have been conducted through different methods for various gynecological conditions in terms of genome, transcriptome, proteome, metabolome, and microbiome since the 1970s (Waldman et al., 1972; Van Ostade et al., 2018; Martínez-Rodríguez et al., 2021).

Traditionally, biological secretions can be used as clues to identify and evaluate the circumstances of the human body. With the rise of omics technologies, the opportunity for the early diagnosis of certain diseases parallelly rises in the use of liquid biopsy assays (Csősz et al., 2017; Janssens et al., 2017). In addition to blood, urine, and cerebrospinal fluid; several other body fluids including tears, nasal mucosa, saliva, sweat, sebum, milk, stool, semen, vaginal mucus, and CVF might be regardable in the screening of certain diseases. From this point of view, several studies have been designed to identify novel biomarkers in different types of body fluids, which have been aided by the favor advances in mass spectrometry analysis (Kulasingam et al., 2008; Horgan et al., 2011; Bandu et al., 2019).

In the case of CVF, this body fluid contains approximately 92%–95% of water and approximately 5%–8% of ions, soluble macromolecules, and proteins (Tsiligianni et al., 2001; Adnane et al., 2018). Among the soluble macromolecules of the CVF, there are DNAs, RNAs, metabolites, proteins, and huge amounts of glycoproteins, proteoglycans, and lipids. The proportions of those components are regulated by physiological circumstances. Many factors can affect the amount, composition, content proportions, and properties of the human cervicovaginal cerclage; not only pathological conditions such as bacterial, fungal, viral infections, or cancer but also menstrual cycle, hormonal imbalance, menopause, fertility/infertility, sexual intercourse, pregnancy and labor, even smoking can alter the CVF composition (Chappell et al., 2014; Adnane et al. 2018; Riganelli et al., 2020).

Currently, more than a thousand different proteins have been identified in CVF and some of these proteins are biomarkers associated with pathological conditions such as vaginal dysbiosis (Kindinger et al., 2016; Hong et al., 2020), amniotic inflammation (Vicente-Muñoz et al., 2020), preterm labor (Elovitz et al., 2019), preeclampsia (Terzi et al., 2015), ovarian cancer (Casado-Vela et al., 2009), endometrial cancer (Shukair et al., 2013), HIV infection (Pattyn et al., 2019), HPV infection (Łaniewski et al., 2019), or CxCa (Van Raemdonck et al., 2014; Starodubtseva et al., 2019).

During the last 20 years, many studies have been conducted to detect changes in the protein expression profiles of CVF in response to different physiological conditions (Martínez-Rodríguez et al., 2021). Mass spectrometry systems are convenient for biomarker discovery and disease screening (Csősz et al., 2017; Bandu et al., 2019) by using biological fluids. Particularly, proteomics approaches have been found to be useful in identifying disease-related diagnostic and prognostic biomarkers (Martínez-Rodríguez et al., 2021). Given that, the CVF represents the carcinogenic tissues for CxCa, is the best source of biomarkers for the evaluation of malignant transformation (Van Ostade et al., 2018). Moreover, although serum, urine, and cytological specimen biomarkers have been widely investigated for HPV infection and CxCa screening, these biological samples are not specific to the transformation area, and may also indicate the presence of other physiological conditions throughout the body. In contrast, the protein composition of the CVF provides an insight into the biochemical pathways involved in pathological alterations of the associated tissue. In this review, the proteome of the CVF is overviewed in terms of its clinical applicability.

## 5. Proteomics studies and potential protein biomarkers from CVF for CxCa

**Figure 4:**
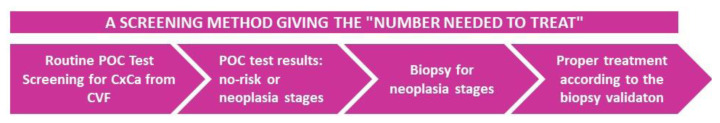
Ideal screening approach of CxCa. To avoid inexact and excessive testing for CxCa screening, a novel screening approach is targeted to capture the only cancer-associated biomarkers, rather than HPV infection. To reveal the number needed to treat increases, and the neoplasia presence can be detected, rather than premalign lesions.

POC devices designed for the detection of the DNA or oncoproteins of the HPV cannot be used for cancer diagnosis and/or prognosis, because HPV infection may not indicate the presence of carcinogenesis. Also, most HPV infections are transient and do not progress to premalignant lesions (Insinga et al., 2007; Shannon et al., 2017) and do not produce true cancer precursor lesions (Figure 2). Besides, HPV positivity usually results in panic and anxiety, especially among uneducated and/or younger women (Qin et al., 2020). An ideal screening strategy should identify high-risk symptoms that may progress to cancer and should incorporate prognosis into the treatment (Figure 4). In this direction, a POC test must be repeatable, reliable, and cancer-associated method rather than being infection-based.

The proteomic studies which aim to evaluate the protein composition of CVF and/or identify candidate protein biomarkers within CVF for detecting premalign stages of CxCa were summarized in Table.

Significant proteins were listed in Table which are upregulated in CVF during carcinogenesis. All of these proteins are not only found in the extracellular secretions’ proteome but also some of them are identified in the intracellular and membrane proteins of localized cells (Van Ostade et al., 2018). Some of these significant proteins may be associated with the cytoskeleton, cytoplasm, or membrane, or they may act as inhibitors, enzymes, or metabolic pathway mediators. Also, those are not specific localized tissue proteins. Nevertheless, a significant increase in the amount of these protein biomarkers in CVF is unique to cervical carcinogenesis. The function of these significant proteins in the development of CxCa was not investigated; however, their significant upregulation in the CVF makes them promising biomarkers, without their functional associations. Forasmuch as cancer biomarkers are released in large amounts when the tissue undergoes neoplastic conversions, and their clinical reliability is heavily dependent on their sensitivity and specificity rates, not their role in carcinogenesis. Our group is currently working on the quantitative validation of some of these candidate protein biomarkers with a large sample biobank to reveal their exact sensitivity and specificity.

As inferential from the studies in Table, the CVF proteome can be divided into two fractions: the core proteome and the variable proteome. The core proteome, including the secretions of the localized healthy microbiome, is robust and highly abundant, while the variable proteome is flexible and at much lower abundance (Shaw et al., 2007; Zegels et al., 2007; Boylan et al., 2014; Parry et al., 2020). The core proteome widely consists of glycoproteins, proteoglycans, and immunoglobulins. Mucin proteins, which are combined with the carbohydrate groups such as sialic acid, play a role in constructing the mucus, and alterations of any member can disrupt the physical properties of the mucus (Adnane et al., 2018) and may affect the local homeostasis. A protein biomarker can be found in both core and variable proteome.

Proteins in high and low abundance may originate from any atypical conditions of the cervicovaginal area; however, artifacts might be generated by differences through the collection method and the degree of disruption of the epithelial cell layer of the cervix. Hence, at this point, the importance of the sample collection method emerges. For instance, the lavage aspiration of the cervicovaginal area has a minimal disruption of the cell layer of the cervix and around. On the other hand, the proteins that were found significant may also vary due to differences in the separation of digestion methods and mass spectrometry systems between studies. Taken together, all of the significant proteins placed in Table must be furtherly validated and verified to evaluate their clinical significance. These studies were designed to index the CVF proteome and some of those compared the healthy and patient groups and identified the candidate biomarkers for the malignant transformation of the cervix. However, as few as three of these studies have performed quantitative validation of their potential biomarkers, aimed at the development of novel CxCa screening tools.

The first of these three studies (Van Raemdonck et al., 2014) is aimed at constructing the differences in the protein expression profiles of CVF in healthy and HPV-infected women with different stages of premalign cervical lesions. To this end, they performed a semiquantitative proteomic study of cervicovaginal lavage samples collected during colposcopic examinations of 6 healthy individuals, 4 patients with LSIL, and 2 patients with HSIL. The proteomic analysis was performed using high-performance liquid chromatography (HPLC) and matrix-assisted laser desorption ionization-time of flight (MALDI-TOF/TOF) mass spectrometry (MS). They found 16 proteins as significant between the groups. They noticed that actinin-alpha-4 (ACTN4; p-value: 0.001) is the most likely biomarker to be suitable for the development of a POC test for CxCa screening. They went on to quantitatively support this hypothesis by performing ELISA on samples from 16 healthy and 12 HPV-infected precancerous samples, additionally. The results of this study confirmed that ACTN4 could be used as a diagnostic marker for CxCa with an AUC value of 86%; sensitivity and specificity values of 84% and 86%, respectively.

The second of these studies, a semiquantitative protein expression analysis, was conducted by Starodubtseva et al. in 2019. This was a label-free quantitative proteomic study designed to reveal the early biomarkers in the neoplastic transformation of the cervix. Cervicovaginal lavage samples were collected during the gynecological examination and the study was divided into two parts. The first part was built with 40 samples, including 24 from healthy individuals, and 18, 16, and 15 from patients with LSIL, HSIL, and CxCa groups, respectively. Samples were analyzed by tandem nano-HPLC-MS/MS. The first part of the study was discovering the novel biomarkers. The second part of the study was built with 33 samples, including 14 from healthy individuals, and 8, 6, and 5 from LSIL, HSIL, and CxCa groups, respectively. Thus, the second part of the study was to validate the initial findings through the ELISA test against the most promising target ACTN4. While the sensitivity and specificity of the first part (including mass spectrometer analysis) were determined as 100% and 100%, which are reduced respectively to 77% and 94% in the second part. Even so, the AUC was 87%, and this study identified six potential proteins as candidate protein biomarkers for the early diagnosis of CxCa, particularly ACTN4.

The third of these quantitative proteomics studies, the identification of candidate protein biomarkers for CIN2+ stages, was conducted by Gutiérrez et al. in 2021. They collected the CVF samples with an endocervical brush onto cotton-based FTA (Flinders Technology Associates) elute microcards and used the filter-based protein digestion method. They established the study with 40 samples for (HPV-negative) the control group, and 59 samples for the patient (infected with the high-risk HPV-positive and cervical histology with CIN2+) group. The peptide separation was performed by RP-LC using a nano-LC system combined with Q-Exactive Plus Orbitrap for MS analysis. They stated that a “predictive 7-protein multivariate model” was developed to discriminate the 2+ neoplasia stages, with 90% of sensitivity and 55% of specificity. However, they did not perform the antibody-based validation, which is frequently required to confirm proteomics results quantitatively (Gutiérrez et al. in 2021). After all, the Western blotting or the ELISA test methods have been seen as complementary to proteomics study results.

Regarding three of these studies, only ACTN4 and ANXA2 proteins are common (Table) as significant in two of them (Van Raemdonck et al., 2014; Starodubtseva et al., 2019). Therefore, in each proteomics study, different proteins can be identified even if they had a similar design in terms of biological samples and/or research methods are used in. At this point, the importance of validation of detecting the target proteins with a quantitative method in an unbiased large sample size emerges.

CVF has great potential as a source for biomarkers for pathologies of the female genital tract. It may yield plenty of information about the functioning of the female genital tract. The proteomic studies may improve the understanding of the microenvironmental changes due to the various physiological conditions mentioned earlier. Nonetheless, the issues about the reliability of the biomarkers and the development of biomarker-based screening tests often depend on their method validation. Potential biomarkers generally cannot survive further validation. The probable reason for this is that although quantitative analyzes were performed to identify the CVF, not much attention has been given to the contribution of its biophysiological and also inter- and intraindividual variation, so far.

Proteomic studies using the latest technology can further expand the CVF proteome and reveal novel candidate biomarkers. This section summarizes the studies that suggest potential candidate protein biomarkers that must be validated and verified by further and larger clinical studies. The development of an algorithm of diagnosis through an immunoassay or integrated multiplex microfluidic cancer biochip with two or three of the protein biomarkers may be the alternative screening method of CxCa. We believe that there is a high probability about such noninvasive novel methods will eventually be replaced with traditional methods.

## 6. Conclusion

CVF may yield information about the health, function, and current prognosis of female genital tract diseases with several parameters such as the microbiome, pH, protein, and metabolite composition of this region. A novel method may be developed by the applications of advanced proteomic analysis and may help expand our understanding of the molecular environmental changes associated with the menstrual cycle, fertility, age, pregnancy and preterm birth, infections, and cancers of the female genitourinary area. Findings of metaanalysis and systematic reviews (Nelson et al., 2017) indicate that self-sampling and screening by which the patient can interpret the result CxCa is acceptable. Patients may prefer self-sampling owing to its ease of use, safety, privacy, and cost-effectiveness (Nelson et al., 2017; Belokrinitskaya et al., 2019). Also, it brings to reach more women for regular CxCa screening throughout the world.

Increased attendance rates that have been tracked through self-sampling (Nelson et al., 2017) promote the self-screening approach. The overall acceptability of self-screening may lower the costs leading to better prevention of CxCa (Belokrinitskaya et al., 2019). A novel screening method prospect discussed in this review could help reduce the CxCa prevalence and can determine the “number needed to treat”, by assessing the risk of cancer instead of HPV infection (Figure 4). This test could help identify the patients who need biopsy intervention at one-step and reduce the overall clinical costs in LMICs. Further clinical validation is required to include the biomarkers in routine clinical practice, and this study serves to highlight the studies for revealing the cancer-associated protein biomarkers which are the best suited for clinical diagnostic and prognostic tests. A single biomarker may not be sufficient for the development of reliable POC tests for CxCa as other gynecological cancers or pathological conditions may be detected by similar clinical symptoms; therefore, the POC tests should be designed to incorporate a multiplex biomarker set.

POC devices should also focus on applying novel biosensing technologies, which may improve reliability and facilitate the use of several protein targets. There have been several significant advances in this field over the last 15 years, which suggests that it may soon be possible to start producing low-cost clinical diagnostic solutions for women around the world.

## Figures and Tables

**Table:** Proteomics studies sampled from cervix or cervicovagina for CxCa.

	Short citation	Sample collection	Separation method	Mass spectrometry	Results	Significant proteins
**1**	Venkataraman et al., 2007	CVF; collected by menstrual cup; from healthy women	2D SDS-PAGE	MALDI-TOF/TOF	Discovery proteomics: 18 polypeptides identified	
**2**	Tang et al., 2007	Cervicovaginal lavage; collected by aspiration; from healthy women	2D SDS-PAGE	MALDI-TOF/TOF	Discovery proteomics: 59 proteins identified	
**3**	Shaw et al., 2007	Cervicovaginal lavage; collected by gauze; from healthy women	SDS-PAGE or SCX-LC and followed by RP-LC	ESI-Linear IT	Discovery proteomics: 685 proteins identified	
**4**	Zegels et al., 2009	Colposcopy washings; collected by aspiration; from HPV-infected women	Ultrafiltration or LC with C4 and then C18 column	MALDI-TOF/TOF	Discovery proteomics: 339 protein identified	
**5**	Boylan et al., 2014	Liquid-based cytological specimen; collected by SurePath; from healthy women	HPLC	LTQ Orbitrap Velos	Discovery proteomics: 714 protein identified; 153 of determined as “healthy core proteome of cervicovaginal area”	
**6**	Van Raemdonck et al., 2014	Colposcopy washings; collected by aspiration; from healthy and HPV-infected at different risks of precancerous women	RP-HPLC with C4 and then C18 column	MALDI-TOF/TOF	Discovery proteomics: 371 proteins identified	ACTN4, SERPINB3, ANXA2, PKM2 PGK1, VTDB, CFAH, YWHAE, CFH, CRABP2, NAMPT, SERPINB13, ACTR3, HP, ATP5F1B, UBIQ
**7**	Borgdorff et al., 2015	Cervicovaginal lavage; collected by aspiration; from women who have various sexually transmitted infections	nanoACQUITY-nLC system	LTQ Orbitrap Velos	Discovery proteomics: 549 proteins identified	
**8**	Starodubtseva et al., 2019	Cervicovaginal lavage; collected by aspiration; from healthy and HPV-infected at different risks of precancerous women	HPLC	LTQ FT MS/MS	Discovery proteomics: 675 proteins identified	ACTN4, ANXA1, ANXA2, VTN, CAP1, MUC5B
**9**	Ma et al., 2020	Cervicovaginal mucus; from healthy, endocervical adenocarcinoma, cervical adenocarcinoma in situ	iTRAQ labeling, Ultimate 3000 HPLC system	TripleTOF LC-MS/MS	Discovery proteomics: 711 proteins identified	
**10**	Gutiérrez et al., 2021	CVF, collected by brush; from healthy and HPV-infected at different risks of precancerous women	C18 column and reversed-phase EASY-nLC system	nano-LC-MS/MS	Discovery proteomics: 3699 proteins identified	RAB6A, RPS27A, TRIM29, PHB2, ARPC5, KARS1, TES

## Abbreviations

2D: Two-dimensional
ACTN4: Alpha actinin-4
AGUS: Atypical glandular cells
ANXA1: Annexin-A1
ANXA2: Annexin-A2
ASC-H: Atypical squamous cells cannot exclude HSIL
ASCUS: Atypical squamous cells of undetermined significance
AUC: Area under curve
CAP1: Adenylyl cyclase-associated protein-1
CIN: Cervical intraepithelial lesion
CVF: Cervicovaginal fluid
CxCa: Cervical cancer
DNA: Deoxyribonucleic acid
ESI-LIT: Electrospray ionization-linear ion trap
HIV: Human immunodeficiency virus
HPLC: High-performance liquid chromatography
HPV: Human papilloma virus
HSIL: High-grade squamous intraepithelial lesion
LAMP: Isothermal loop-mediated amplification
LC: Liquid chromatography
LFA: Lateral flow assay
LMICs: Low and middle-income countries
LSIL: Low-grade squamous intraepithelial lesion
LTQ: Linear trap quadrupole
LTQ-FT-MS/MS: Linear trap quadrupole Fourier transform mass spectrometry
MALDI-TOF/TOF: Matrix-assisted laser desorption ionization time of flight
MS: Mass spectrometry
MUC5B: Mucin-5B
nLC: Nanoscale liquid chromatography
Pap-smear: Papanicolaou stain cervical smear test
POC: Point-of-care
RNA: Ribonucleic acid
ROC: Receiver operating characteristic
RP: Reverse phase
SCX: Strong cation-exchange chromatography
SDS-PAGE: Sodium dodecyl sulphate-polyacrylamide gel electrophoresis
VTN: Vitronectin
WHO: World Health Organization
